# Kinetics of neutralizing and binding antibody responses following Zika virus infection during pregnancy: A nested analysis of participants from the microcephaly epidemic research group (MERG) and Zika in infants and pregnancy (ZIP) cohorts

**DOI:** 10.1016/j.virusres.2026.199725

**Published:** 2026-04-05

**Authors:** Maria Valquíria de Medeiros Silva, Ricardo Arraes de Alencar Ximenes, Demócrito de Barros Miranda Filho, Celina Maria Turchi Martelli, Thalia Velho Barreto de Araújo, Elizabeth Brickley, Juliana Menezes Soares de Souza Azevedo Fontes, Gabriela Renata Neves Fulco, Ana Carolyne de Carvalho Lucena Sá, Marli Tenorio Cordeiro, Priscila Mayrelle Da Silva Castanha, Paula Alexandra dos Santos Oliveira, Ulisses Ramos Montarroyos

**Affiliations:** a Programa de Pós-graduação em Ciências da Saúde, Universidade de Pernambuco, Av. Governador Agamenon Magalhaes 2110, Recife, Pernambuco 50100-250, Brazil; b Faculdade de Ciências Médicas, Universidade de Pernambuco, Av. Governador Agamenon Magalhaes 2110, Recife, Pernambuco 50100-250, Brazil; c Departamento de Medicina Tropical, Universidade Federal de Pernambuco, Av. Professor Moraes Rego, 1235, Cidade Universitária, Recife, Pernambuco 50670-901, Brazil; d Departamento de Saúde Coletiva, Instituto Aggeu Magalhães, Fiocruz/PE, Av. Moraes Rego, Campus da Universidade Federal de Pernambuco, Cidade Universitária, Recife, Pernambuco 50670-420, Brazil; e Departamento de Medicina Social, Universidade Federal de Pernambuco, Av. Professor Moraes Rego, 1235, Cidade Universitária, Recife, Pernambuco 50670-901, Brazil; f Department of Infectious Disease Epidemiology, London School of Hygiene & Tropical Medicine, Keppel Street, London WC1E 7HT, United Kingdom; g Departamento de Virologia, Instituto Aggeu Magalhães, Fiocruz/PE, Av. Moraes Rego, Campus da Universidade Federal de Pernambuco, Cidade Universitária, Recife, Pernambuco 50670-420, Brazil; h Department of Infectious Diseases and Microbiology, University of Pittsburgh School of Public Health, 2138 Public Health, 130 DeSoto Street, Pittsburgh, Pennsylvania 15261, USA; i Departamento de Parasitologia, Instituto Aggeu Magalhães, Fiocruz/PE, Av. Moraes Rego, Campus da Universidade Federal de Pernambuco, Cidade Universitária, Recife, Pernambuco 50670-420, Brazil; j Instituto de Ciências Biológicas, Universidade de Pernambuco, Av. Governador Agamenon Magalhaes 2110, Recife, Pernambuco 50100-250, Brazil

**Keywords:** Zika virus, Serology, Pregnant women, Antibodies

## Abstract

ZIKV infection during pregnancy can result in Congenital Zika Syndrome (CZS) in the newborn. Nevertheless, diagnosing ZIKV infection during pregnancy remains challenging, and the kinetics of the serological response to acute infection remains insufficiently reported in the literature. Using samples collected longitudinally from two cohorts of pregnant women in Brazil, this study aims to estimate the timeframe to reach anti-ZIKV immunoglobulin (IgM) negativity. Additionally, it seeks to describe the positivity of IgM, IgG3, and neutralizing antibodies based on the interval between symptom onset and testing in symptomatic pregnant women. In the cohort of pregnant women who tested ZIKV IgM-positive, the probability of IgM seroreversion after the initial detection. was 40% by 3 months, 75% by 6 months, and 85% by 12 months. Moreover, we observed a decreasing trend in the percentage of ZIKV-positive women with detectable ZIKV-specific IgM antibodies over the 12 months after infection, peaking at 8.1% 1 to 2-months after symptom onset and declining to 1.1% by 4 to 5-months after symptom onset. In contrast, IgG3 and neutralizing antibody detection remained stable over the observation period, with approximately <6% and 50% remaining detectable, respectively. This study adds to the body of evidence on the behavior of the antibody responses in a population of women infected with ZIKV during pregnancy. The findings advance understanding of the probability of seroconversion and seroreversion using different immune markers in ZIKV-infected pregnant women, and have the potential to improve the design of maternal screening and surveillance strategies for ZIKV.

## 1 Introduction

A surge in cases of microcephaly in Brazil between 2015 and 2016 prompted the World Health Organization (WHO) to declare a Public Health Emergency of International Concern (Araújo et al., 2016; Albuquerque et al., 2018). As part of the rapid scientific response in Brazil, the Microcephaly Epidemic Research Group (MERG) launched an investigation in January 2016 to identify the cause of the epidemic in Recife, in the Northeastern state of Pernambuco, the epicenter of the epidemic. Through a case-control study, MERG identified an association between the cases of microcephaly and intrauterine Zika virus (ZIKV) infection (Araújo et al., 2018).

ZIKV, a species of the *Flavivirus* genus of the *Flaviviridae* family, was first detected in Brazil in April 2015 (Zanluca et al., 2015). In addition to mosquito vectors, ZIKV transmission may also occur through sexual contact and transfusions and congenitally through the placenta (Silva and Souza, 2016). Although ZIKV infection symptoms (i.e., rash, itching, fever, myalgia, and conjunctivitis) are relatively mild in the general population, infection during pregnancy can lead to fetal infection and disrupt development, resulting in a series of structural and functional defects, clinically recognized as Congenital Zika Syndrome (CZS). In addition to microcephaly, CZS is also associated with imaging, neurological, and ophthalmological abnormalities, congenital Zika manifestations also include imaging, neurological, and ophthalmological abnormalities. In a cohort of pregnant women exposed to ZIKV, the absolute estimated risk among children was 28.3% for developing at least one of these manifestations associated with CZS (Ximenes et al., 2021). In a meta-analysis of offspring of 1548 women with RT-PCR-confirmed ZIKV infections during pregnancy participating in the 13 cohorts of the Zika Brazilian Cohorts Consortium, Ximenes et al. (2023) noted that adverse outcomes resulting from ZIKV infection can occur at any gestational period; however, more severe structural brain abnormalities have been associated with ZIKV infection during early pregnancy (Ximenes et al., 2023).

Following an incubation period of 3 to 14 days, ZIKV infections have a brief viremia period, during which viral particles can be detected in bodily fluids. ZIKV RNA is typically detectable in the blood for up to 7 days (Musso and Cao-Lormeau, 2014), while in urine, it may be detected for more than 10 days. Concurrently, the humoral immune response begins to produce acute-phase antibody, Immunoglobulin M (IgM), which may appear in serum 2 to 3 days after the onset of symptoms (Priyamvada et al., 2017), and can thereafter be detected for up to 3 months (Sharma and Lal, 2017). Immunoglobulin G (IgG) begins to become detectable in serum from 7 to 8 days after the symptom onset, and peaks between 15 and 30 days, and may persist for months to years, while IgM levels wane (Sharma and Lal, 2017).

However, the literature presents differing reports regarding both viremia and the antibody response in ZIKV-infected pregnant women. Prolonged viremia has been documented in one pregnant woman in whom the virus remained present in serum samples for 107 days after symptoms (Suy et al., 2016). Similarly, there was also the case of a pregnant woman with viremia for 70 days, until the pregnancy was terminated (Driggers et al., 2016). Pomar et al. (2021) identified the occurrence of prolonged viremia in a cohort of pregnant women in French Guiana. Among 33 women who tested positive for ZIKV by RT-PCR, 14 exhibited prolonged viremia (defined as > 30 days). In this study, prolonged viremia was associated with a seven-fold increase in the risk of adverse fetal and neonatal outcomes (Pomar et al., 2021).

With regard to the anti-ZIKV antibody response, a study of pregnant women with rash in the state of Pernambuco found that 73 out of 127 women with a positive qRT-PCR test for ZIKV demonstrated no serological evidence of ZIKV infection using enzyme-linked immunosorbent assay (ELISA) test (Ximenes et al., 2019). Ninety days after symptom onset, only 11 (10.5%) of the 105 women with confirmed infection by qRT-PCR presented with seroconversion IgM). Another unexpected finding was that five women with molecularly confirmed infection tested positive for IgM >90 days after the onset of rash (Ximenes et al., 2019).

Given the significant health risks associated with ZIKV infection during pregnancy, pregnant women and those of reproductive age constitute the most vulnerable population to ZIKV epidemics. Therefore, developing reliable methods for the screening and diagnosis of ZIKV in symptomatic and asymptomatic pregnant women remains a key priority for the epidemic preparedness agenda (Qiao et al., 2021). Nevertheless, evidence on the behavior of ZIKV infection markers in pregnant women still requires further clarification (Ximenes et al., 2023), and an improved understanding would facilitate the confirmation of cases of current, recent, or past ZIKV infection in pregnant women. Rapid diagnostic tests for detecting ZIKV circulation within a region enable early public health intervention, thereby reducing the risks of another epidemic, infections during pregnancy, and consequently, preventable developmental delays in children (Ximenes et al., 2023). Accordingly, this study has aimed to establish the timeframe for the IgM infection marker for ZIKV to become negative in seropositive pregnant women, and to characterize the positivity of IgM, IgG3, and neutralizing antibodies in symptomatic pregnant women, according to the interval between symptom onset and testing.

## 2 Material and methods

### 2.1 Study design

This study analyses anti-ZIKV binding and neutralizing antibody responses in serum samples collected longitudinally from two prospective cohort studies conducted in Pernambuco, Brazil: (1) pregnant women who presented with a rash during pregnancy (MERG Pregnant Women’s Cohort, MERG PWC), (2) pregnant women who tested positive for ZIKV by IgM during pregnancy (Zika in infants and pregnancy, ZIP Cohort)."

#### 2.1.1 Inclusion and exclusion criteria for the current study

For the MERG PWC**:** Pregnant women were included in the current study if they underwent IgM, IgG3, and PRNT testing for ZIKV infection. For the ZIP Cohort: Pregnant women were included if they tested positive for ZIKV by IgM. Those with only one test during the cohort period were excluded.

### 2.2 Study place and population

The MERG led the recruitment and follow-up of both cohorts (MERG PWC and ZIP Cohort). While the MERG PWC extended recruitment to a radius of up to 120 km from the Metropolitan Region of Recife, the ZIP Cohort exclusively recruited pregnant women residing in Recife.

**MERG PWC Design:** Pregnant women presenting with a rash at any stage of gestation and with laboratory evidence of ZIKV infection were included in the study. Laboratory evidence was defined as positivity for at least one of the following tests: qRT-PCR, IgM, IgG3, or PRNT. Recruitment took place from May 2016 to June 2017 and was initiated during the visit of the pregnant women to referral health services for prenatal care or other medical services. Healthcare professionals recorded notification on the platform of the Pernambuco Strategic Information Center for Health Surveillance (CIEVS/PE) of the State Health Department, using the FormSUS (available at: cievspe.com). Following notification, public health professionals collected the first biological sample (i.e., blood) during the acute phase, either on the consultation day for the rash or over the following days. The MERG team conducted subsequent blood collections, with the second collection occurring at least 14 days after the first. The third collection took place after childbirth. The time intervals between collections varied among the women, and not all diagnostic tests were performed in each assessment. The collected blood samples were sent to the Laboratório Central de Saúde Pública de Pernambuco (LACEN-PE) (Recife, Pernambuco) where serum samples were separated. Aliquots were sent to the Virology Department of the Aggeu Magalhães Institute, Oswaldo Cruz Foundation-PE (IAM-FIOCRUZ-PE, Recife, Pernambuco), and stored at −80 °C for diagnostic testing.

**ZIP Cohort Design:** The inclusion criterion for this cohort were pregnant women in the first trimester of pregnancy or at the beginning of the second (i.e., up to 17 weeks and 6 days of gestation), who were aged 15 years or older. Pregnant women linked to the Unidade Básica de Saúde (UBS) in the city of Recife were recruited from October 2016 to September 2018. Initially, pregnant women were interviewed for information on their medical history, vaccinations, and pregnancy. Follow-up involved monthly visits throughout the pregnancy, during childbirth, and six-weeks postpartum. For women who tested positive during this period, follow-up was extended to the point when IgM became undetectable. Sequential blood collections were conducted at each visit to assess seroconversion IgM (i.e., defined as a change from negative (< or =2) to positive (>3) status in laboratory testing for IgM detection). regardless of the presence of symptoms. For participants who developed symptoms during follow-up an additional blood sample was collected for testing, and a symptom questionnaire was administered. In cases of seroconversion, pregnant women with a positive result underwent qRT-PCR testing on the blood sample obtained prior to the first positive IgM result. The Virology Department of IAM-FIOCRUZ-PE, (Recife, Pernambuco) was responsible for receiving, processing and storing all blood samples.

### 2.3 Laboratory procedures

**In the MERG PWC:** Serum samples were analyzed for the detection of Zika virus-specific IgM antibodies using an IgM capture enzyme-linked immunosorbent assay (MAC ELISA) based on a protocol from the U.S. Centers for Disease Control and Prevention (CDC, Fort Collins, Colorado, USA). Additionally, samples were also tested for Zika virus-specific non-structural protein 1 (NS1) IgG3 antibodies using an in-house ELISA, which is designed to assesses ZIKV exposure over the previous six months.

For the IgM capture assay, maternal sera were tested in parallel using ZIKV and dengue virus (DENV) antigens to assess for immune cross-reactivity between the closely related flaviviruses. ZIKV-specific neutralizing antibodies were assessed in all available maternal sera using the plaque reduction neutralization test (PRNT). The PRNT50 values were determined as the PRNT endpoint at which the highest serum dilution inhibited >50% of the plaques, according to a previously described modified protocol (Cordeiro et al., 2016). The tests were performed on Vero cells seeded at a density of 300,000 cells/mL in 24-well plates. The test was initiated after serial dilution (dilutions, 1:20 to 1:20 480) of heat-inactivated serum samples at 56 °C for 30 min, followed by incubation with 100 previously titrated plaque-forming units (PFU) (Castanha et al., 2013). For the PRNT, the ZIKV was isolated in Pernambuco and molecularly identified as ZIKV, Brazil-PE243/2015 (Cordeiro et al., 2016). Through a four-parameter non-linear regression, neutralizing antibody levels were estimated, which determined the dilution capable of achieving a 50% reduction in the plaque count (PRNT50). Maternal sera with PRNT50 titers >20 were classified as non-negative for ZIKV, titers PRNT50 >20 and <100 were classified as indeterminate, and titers PRNT50 >100 were considered positive (Ximenes et al., 2019).

**In the ZIP Cohort:** Serum samples were subjected to the MAC ELISA serological assay to detect IgM antibodies for Zika and Dengue (DENV) viruses, using a protocol standardized by the CDC. The MAC ELISA kits were provided by the US National Institutes of Health from BEI Resources (United States). As in the MERG Pregnant Women’s Cohort, tests were performed for both ZIKV and DENV to identify the occurrence of cross-reactivity. The laboratory team produced the DENV antigen from samples of DENV1, 2 and 3 from Laboratório Central de Saúde Pública de Pernambuco (LACEN-PE) and DENV4 from the Evandro Chagas Institute, MS (Belém, Pará), following the protocol used in the production of the ZIKV antigen (CDC). The investigation of antibodies for DENV-IgG (i.e., to detect a previous infection) was conducted using the ALERE-PanBio indirect ELISA Anti-Dengue IgG commercial kit (PANBIO, Inverness Medical Innovations Australia Pty Ltda), according to the manufacturer's instructions.

### 2.4 Statistical analysis

The collected data were analyzed using STATA, version 14.0 (College Station, TX, USA). The characteristics of pregnant women in both cohorts were described. Different analysis methods were applied to each cohort due to their unique data collection strategies.

For the MERG PWC: Blood samples were collected at different time intervals, and there was not necessarily a sequence of samples from the same pregnant woman. Cross-sectional assessments were conducted repeatedly within the cohort. Therefore, the prevalences of positive IgM, IgG3, and neutralizing antibodies, with 95% confidence intervals (CI), were calculated in time strata related to the period between the notification date of the rash (i.e., symptom onset) and the date of blood collection.

For the ZIP Cohort: Since multiple blood samples were collected from the same pregnant woman over time, a survival analysis was conducted to estimate the probability of IgM negativity over time. The time at risk was defined as the period between the date of the positive IgM test and the date of IgM negativity (event) or the end of follow-up (censoring). The incidence rate of IgM negativity per 100 person-months was reported, and the Kaplan-Meier estimator was used to calculate the probability of IgM survival time. The incidence rate was calculated by dividing the number of IgM negative pregnant women by the total time at risk, then multiplied by 100.

### 2.6 Ethical considerations

This study was developed following approval by the Research Ethics Committee of the HUOC/PROCAPE Hospital Complex and has the Certificate of Presentation for Ethical Assessment (CAAE) No. 70198223.0.0000.5192 and a substantiated report No. 6.209.246 issued on July 31, 2023. The Research Ethics Committee of the IAM-Fiocruz-PE provided ethical approval for the MERG Pregnant Women’s Cohort (PWC) under CAAE: 5324.0816.4.0000.5190 and a substantiated report No 1.533.226 issued on May 6, 2016 and for the Zika in Infants and Pregnancy (ZIP) Cohort under CAAE: 5667.3616.3.2001.5190 and a substantiated report No 1.697.660 issued on 26 August 2016. In addition to having guaranteed the standard procedures recommended by Resolution MS/CNS 466/2012, informed consent was obtained from all participants prior to enrollment.

## 3 Results

The MERG Pregnant Women’s Cohort comprised 694 participants. At the first blood sampling, 466 women underwent serological testing for Zika virus (ZIKV), including IgM (*n* = 466), IgG3 (*n* = 175), and PRNT (*n* = 182) assays; the remaining 228 women did not undergo laboratory screening during this phase. In the second follow-up, 688 samples were collected, all of which were tested for IgM, while 211 and 568 samples were analyzed for IgG3 and PRNT, respectively. The third collection yielded 476 samples, with testing performed for IgM (*n* = 476), IgG3 (*n* = 175), and PRNT (*n* = 329) (Fig. 1.A). In the ZIP cohort, 62 pregnant women with positive ZIKV-specific IgM were followed up (Fig. 1.B).

The mean age of the MERG Pregnant Women’s Cohort at enrollment was 25 years, and for the ZIP Cohort, 26.5 years. The majority of participants in both cohorts identified as being Black (*Preta*), Mixed race (*Parda*), or from another racially minoritized background, reported having had a previous pregnancy, were non-smokers, and did not use recreational drugs. Education levels differed between the cohorts: in the MERG Pregnant Women’s Cohort, 44.2% had between 10 and 12 years of education, while 75.8% of the ZIP Cohort fell into this educational category (Table 1).

**Table 1:** Characteristics of the women in the MERG PWC and ZIP cohorts, in the state of Pernambuco, Brazil (2016–2018).

Variables	MERG PWC) (*n* = 694)	ZIP cohort (*n* = 62)
Age in years, Median (IQR)	25.0 (21 to 31)	26.5 (23 to 32)
Race/ethnicity^⁎⁎^		
White (*Branca*)	163 (23.5%)	19 (30.6%)
Black (*Preta*), Mixed race (*Parda*), Asian, Indigenous, or other	531 (76.5%)	43 (69.4%)
Years of education		
0–9	313 (45.0%)	9 (14.5%)
10–12	306 (44.2%)	47 (75.8%)
13+	75 (10.8%)	6 (9.7%)
Previous pregnancy		
Yes	435 (62.5%)	42 (67.7%)
No	259 (37.5%)	20 (32.3%)
Smokers		
Yes	62 (9.0%)	1 (1.6%)
No	632 (91.0%)	61 (98.4%)
Use of recreational drugs		
Yes	14 (2.0%)	0 (0%)
No	680 (98.0%)	62 (100%)

### 3.1 Results of the successive cross-sectional studies of the MERG PWC

Table 2 present the prevalence of IgM, IgG3 positivity, and neutralizing antibodies across different time strata. Each time stratum represents the interval between the onset of symptoms and the blood collection. Thus, the analysis was based on the quantity of tests performed per period, which differs from the number of pregnant women participating.

**Table 2:** IgM and IgG3 positivity, and neutralizing antibodies over time - MERG PWC, in the state of Pernambuco, Brazil (2016–2017).

Time interval (symptoms and blood collection)	(IgM) n/total tests (% - CI95%)	IgG3 n/total tests (% - CI95%)	Neutralizing antibodies (PRNT) n/total test (% - CI95%)
Up to 1 month	34/454 (7.5%; 5.4 to 10.3)	10/172 (5.8%; 3.1 to 10.5)	72/186 (38.7%; 31.9 to 46.0)
> 1 to 2 months	14/171 (8.2%; 4.9 to 13.4)	3/77 (3.9%; 1.2 to 11.6)	71/131 (54.2%; 45.5 to 62.6)
> 2 to 3 months	9/175 (5.1%; 2.7 to 9.6)	2/68 (2.9%; 0.7 to 11.4)	79/160 (49.4%; 41.6 to 57.2)
> 3 to 4 months	5/187 (2.7%; 1.1 to 6.3)	2/55 (3.6%; 0.9 to 13.9)	82/171 (47.9%; 40.5 to 55.5)
> 4 to 5 months	1/90 (1.1%; 0.2 to 7.7)	5/36 (13.9%; 5.6 to 30.3)	37/75 (49.3%; 38.0 to 60.7)
> 5 to 6 months	1/66 (1.5%; 0.2 to 10.5)	1/17 (5.9%; 0.7 to 37.2)	23/48 (47.9%; 33.8 to 62.3)
> 6 months	13/449 (2.9%; 1.7 to 4.9)	5/117 (4.3%; 1.8 to 10.0)	118/287 (41.1%; 35.5 to 46.9)

Thus, it was identified that the prevalence of IgM positivity begins to decrease between the second and third months, with lower levels observed from the fourth month onwards, and according to the CI, is statistically significant between the periods "up to 1 month" and "> 6 months". The prevalence of IgG3 positivity and neutralizing antibodies remained relatively constant throughout the observation period. The maximum timeframe of IgM positivity was up to 17 months. Of the 694 pregnant women, 125 (18%) were tested for dengue (IgG), of whom 93 (94.4%) presented positive results.

### 3.2 Results of the survival analysis in the ZIP cohort

Table 3 presents the characteristics of the 62 IgM-positive participants in ZIP cohort, who underwent at least two serological tests for IgM detection during a median follow-up of 3.5 months and a total time at risk of 313.3 months.

**Table 3:** Description of IgM testing in the ZIP cohort in the state of Pernambuco, Brazil (2016–2018).

Characteristics of the cohort	Statistics
Number of participants with at least two or more IgM tests	62
Number of IgM tests during follow-up^a^	4 (2 to 15)
Number of subjects whose IgM became negative during follow-up	51 (82.3%)
Follow-up time (in months)^a^	3.5 (0.5 to 26.6)
Total follow-up time (in months)	313.3
Estimated time to IgM negativity (*n* = 51) (in months)^a^	3.5 (1.2 to 26.6)
Incidence rate of IgM negativity (100 person-months) (CI 95%)	16.3 (12.4 to 21.4)
Quartiles of time to negativity (in months)	
25%	1.8
50%	3.6
75%	6.5

a Median (Minimum – Maximum).

Of the 62 participants who at some point had IgM detected in the serological tests, 51 (82.3%), subsequently presented a negative IgM during follow-up. The estimated median time to IgM negativity was 3.5-months, and the incidence rate of IgM negativity was 16.3 per 100 person-months. Notably, 25% of pregnant women achieved a negative IgM within 1.8 months, 50% within 3.6 months, and 75% within 6.5 months. All participants were tested for the detection of previous DENV infection through IgG, and 61 of 62 (98.4%) had positive results.

Figs. 2.A.i, Fig. 2Aii, Fig. 2B illustrates the behavior of IgM antibody levels over time for each individual. There was a mean increase in IgM levels during the first 15 days post-positivity, followed by a decline. Approximately 3 months after positivity, the average IgM antibody level became negative (titer <3).

Considering IgM negativity as the event of interest in the survival analysis, the Kaplan-Meier survival curve (Fig. 3) shows the approximate probability of IgM seroreversion was 40% within 3 months was 40%, and 75% within 6 months. By 12 months, the probability of a woman’s samples testing IgM-negative was 85%.

**Fig. 1A:**
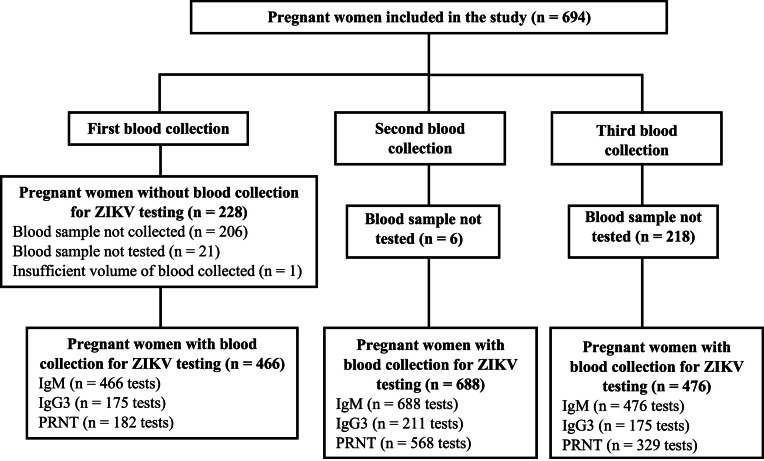
caption.

**Title:** Flowchart of laboratory tests performed in the MERG PWC. **Description:** Flowchart illustrating the sequence of laboratory tests conducted in the MERG Cohort of pregnant women in Pernambuco, Brazil, during the period 2016–2017.

**Fig. 1B:**
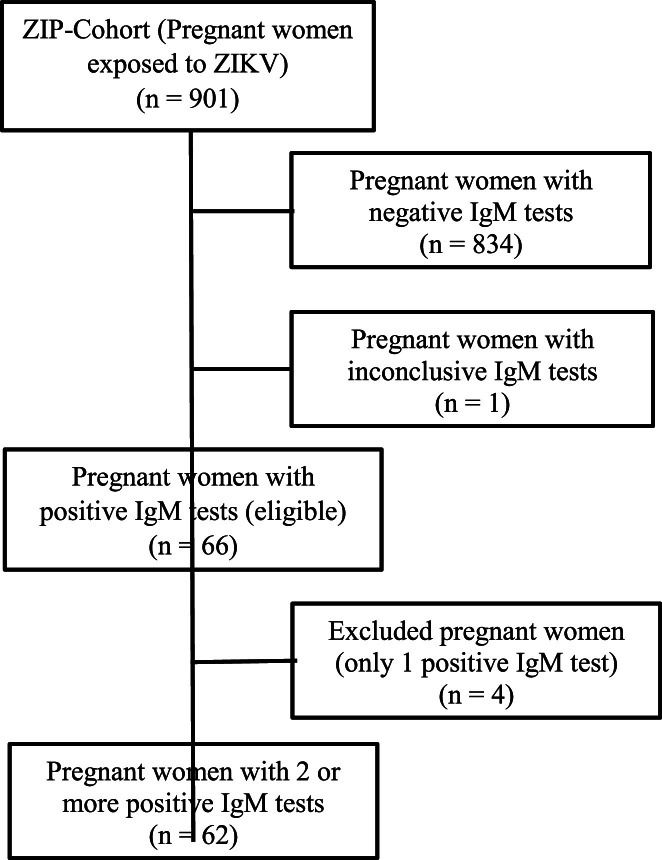
caption.

**Title:** Flowchart of laboratory tests performed in the ZIP Cohort. **Description:** Flowchart illustrating the sequence of laboratory tests conducted in the ZIP Cohort of pregnant women in Pernambuco, Brazil, during the period 2016–2018.

**Fig. 2Ai:**
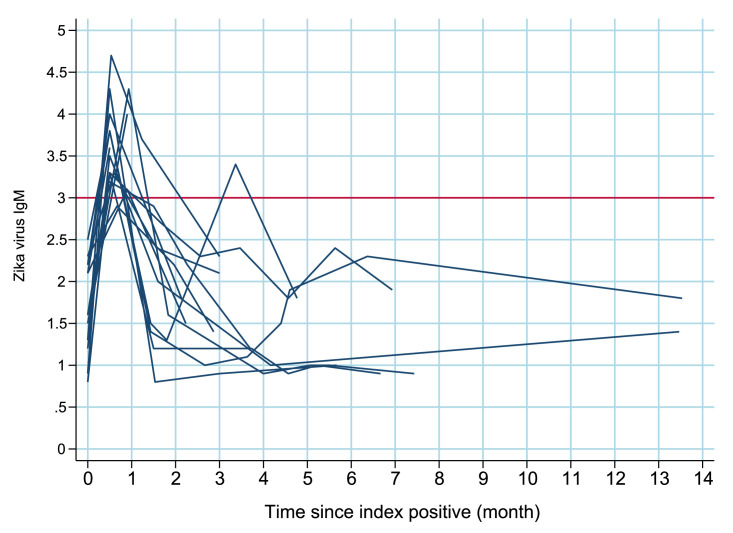
caption.

**Title:** Trajectories of IgM antibodies against Zika virus in pregnant women who tested negative within 3 months: ZIP Cohort, Pernambuco, Brazil (2016–2018). **Description:** Projections of trajectories of IgM antibodies against the Zika virus in pregnant women who tested negative within 3 months from the ZIP Cohort, conducted in Pernambuco, Brazil, between 2016 and 2018.

**Fig. 2Aii:**
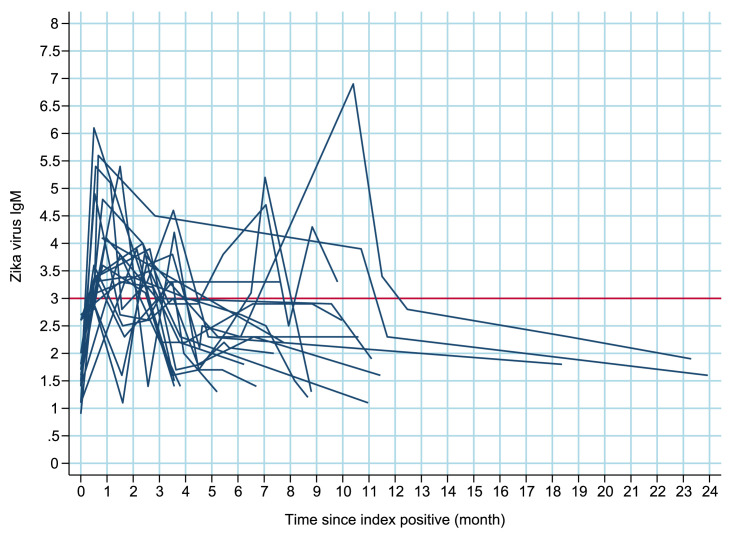
caption.

**Title:** Trajectories of IgM antibodies against the Zika in pregnant women who tested negative after 3 months: ZIP Cohort, Pernambuco, Brazil, between 2016 and 2018. **Description:** Projections of trajectories of IgM antibodies against the Zika virus in pregnant women who tested negative after 3 months from the ZIP Cohort, conducted in Pernambuco, Brazil, between 2016 and 2018.

**Fig. 2B:**
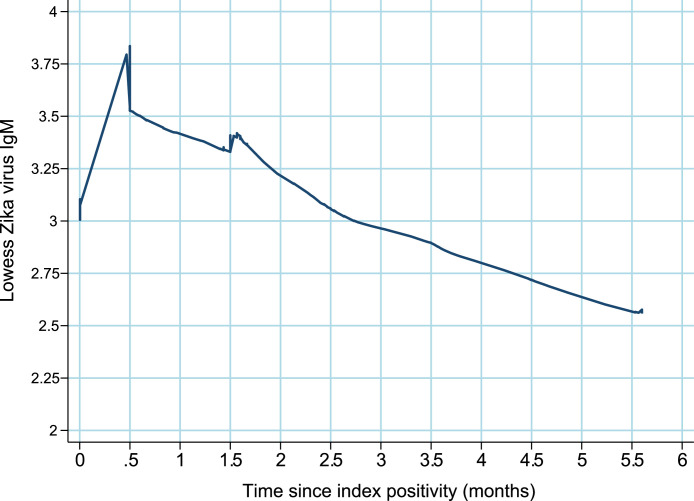
caption.

**Title:** Smooth trend line (lowess) IgM antibodies trajectory for the Zika virus in pregnant women from the ZIP Cohort, Pernambuco, Brazil, between 2016 and 2018. **Description:** Smooth trend line (lowess) IgM antibodies trajectory for the Zika virus in pregnant women from the ZIP Cohort, followed in Pernambuco, Brazil, between 2016 and 2018.

**Fig. 3:**
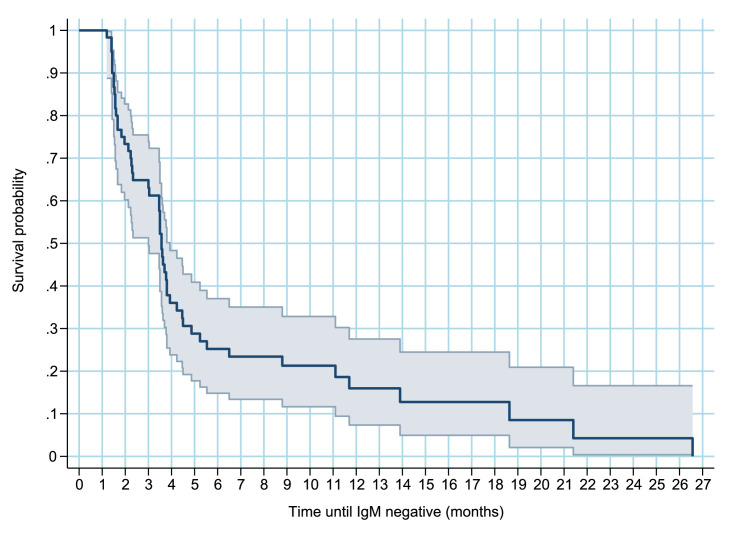
caption.

**Title:** Kaplan-Meier probability of persistence of anti-ZIKV IgM antibodies among pregnant women in the ZIP Cohort, Pernambuco, Brazil, between 2016 and 2018. **Description:** Kaplan–Meier survival curve showing the probability of persistence of anti-ZIKV IgM antibodies among pregnant women in the ZIP Cohort, followed in the state of Pernambuco, Brazil, between 2016 and 2018.

## 4 Discussion

This study has provided relevant and relatively underexplored information in the literature regarding the antibody response in pregnant women with ZIKV infection, a population that may present unique immunological characteristics compared to the general population. In addition, pregnant women constitute the group at greatest risk for this viral infection, thereby underscoring the urgent need to understand their defense mechanisms.

In the survival analysis conducted on the ZIP Cohort, 82.3% (51/62) of pregnant women tested negative for IgM during follow-up, with an estimated median time to IgM negativity of 3.5 months. The mean IgM antibody level decreased to below the negativity cutoff after three months. A downward trend was observed after three months, with a progressive decrease noted in the level of antibodies measured. The probability of IgM negativity was 40% by 3 months, 75% by 6 months, and around 85% by 12 months. Optical density (i.e., indicating the relative concentration of IgM in serum) presented an average increase in the first 15 days after the first positive test. These findings align with those of Stone et al. (2019), who used survival analysis to identify seroreversion through IgM anti-ZIKV detection tests. Among 43 blood donors followed for more than six months, 34 (79%) tested negative (Stone et al., 2020). Another point that corroborates our findings was the persistence of ZIKV IgM detection for >12 months in only a few cases; (Stone et al., 2020) in the ZIP Cohort, the probability of maintaining IgM anti-ZIKV positivity after 12 months was around 15%.

In the analysis of successive cross-sections referring to the MERG Cohort, a decrease in the prevalence of positive IgM was observed between the 2nd and 3rd months (5.1%) with lower values from the 4th month (1.1%). In the IgG3 detection tests, which indicates a recent infection, and in the detection of neutralizing antibodies through PRNT, the prevalence was approximately constant over time for both markers. This pattern differs from the findings of Nascimento et al. (2018), who reported a decline in IgG3 levels between the fourth and sixth months for dengue. The persistence of IgG3 for longer than six months in our study limits its utility as a marker of recent Zika infection. In a study by Hoen et al. (2019), who investigated the behavior of anti-ZIKV antibodies in 60 infected pregnant women confirmed by the RT-PCR test in Guadeloupe, France, it was identified that after 62 days of symptoms, only 7% presented a positive IgM (Hoen et al., 2019). Although this study performed a smaller number of tests compared to the present study, it is observed that the prevalence of seropositivity is low, suggesting that the seroconversion rate was low, which is corroborated by the MERG study, which discovered seroconversion of only 10.5% who were RT-PCR-positive for ZIKV (Ximenes et al., 2019).

Another study investigated the behavior of antibodies to ZIKV by analyzing 300 serum samples obtained from French Guiana and collected up to 300 days post-symptom onset from 124 patients with arboviral-like syndrome, and RT-PCR-confirmed ZIKV infection. Two serological assays were used: the Euroimmun test, which detected IgM in 71% of serum samples collected between 15 and 30 days after symptom onset, in <9% between 61 and 90 days, and in 4% more than 180 days after symptoms. With the Dia.Pro Zika virus IgM test demonstrated detection rates over the same time intervals of 93%, 38%, and 29%, respectively (Matheus et al., 2019). Despite variations in test performance, both sets of results align with our study, demonstrating a reduction in seropositivity after 60 days post-symptom onset.

In contrast, Griffin et al. (2019) observed the persistence of IgM detection in 73% (45/62) of individuals residing in Miami-Dade County, Florida, USA, with laboratory-confirmed ZIKV infection through molecular testing, with follow-up periods ranging from 12 to 19 months after symptoms (Griffin et al., 2019). It is important to note that neutralizing antibodies for DENV were detected in 63% of participants. The persistence of IgM prevalence identified in a group of individuals that excluded pregnant women diverges from studies conducted with this population, corroborating with the existence of specificities in biomarker behavior in ZIKV infection in pregnant women. This underscores the need for further research to broaden our knowledge in this area.

The values for detecting neutralizing antibodies to ZIKV in the MERG Cohort were higher than those obtained in a study in two communities in southern Thailand following the ZIKV outbreak, in which the seroprevalence in the two communities among pregnant women was 24.3% in community A and 12.8% in community B (Densathaporn et al., 2020). The divergent values may be attributed to the timing of applying the PRNT test. In this study, the test was applied just 18 months after the outbreak, whereas in the MERG cohort it was applied from the first month after symptom onset, with monthly follow-ups until the 6th month or later. However, both studies highlight the presence of infection markers over time through the detection of neutralizing antibodies using the PRNT technique. Furthermore, these findings indicate that the population has not reached the theoretical herd immunity threshold of 85.5% (Densathaporn et al., 2020). These data underscore the ongoing risk in the population, especially in pregnant women, who face the most severe consequences of ZIKV infection.

While previous DENV infection has been associated with potential interference in the immune response to ZIKV infection (Stone et al., 2020), we were unable to conduct a similar analysis in our study. This was due to the high prevalence of DENV IgG antibodies among the pregnant women, justified by the fact they lived in a region endemic for flaviviruses. It should be noted that individuals with prior DENV infection have been documented as lacking a IgM response, while exhibiting high levels of IgG against ZIKV at the time of diagnosis. This immune response may be related to the structural similarities between the proteins of DENV and ZIKV, which can lead to antibody cross-reactivity, and potentially inhibiting or reducing the immune response to antigens similar to the those to which they were previously exposed (Barzon et al., 2018).

To date, few other studies (Mor et al., 2017) have examined the kinetics of the anti-ZIKV antibody response in pregnant populations with pre-existing DENV-specific immunity, which has made it difficult to contextualize our findings. It should be noted that immunological changes occur in pregnant women during gestation (Mor et al., 2017) however, it is unclear whether there are differences in the immune response to ZIKV infection compared to the general population. Another key point concerns laboratory testing: while the serological tests applied detect ZIKV infection, cross-reactivity may also occur, since this is an endemic region with simultaneous circulation of other flaviviruses, such as DENV. Although we used the PRNT test for diagnostic confirmation, considered gold standard for diagnosing primary ZIKV infection, there remains the possibility of ZIKV-DENV antibody cross-reaction in individuals with a previous DENV infection.

## 5 Advantages and limitations

The advantages of this study include the focus on pregnant women, a population of particular interest given the need for more accurate diagnostics in this group. Despite concerted efforts by the scientific community, significant gaps in understanding this area remain. Furthermore, our study incorporated data from two cohorts, enabling follow-up during and after pregnancy. A key advantage of our study was our ability to go beyond IgM detection, observing its trajectory over time until negativity was reached. It is also important to note that we used the primary molecular and serological laboratory tests, which provided a comprehensive understanding of the immune response. By confirming infection via RT-PCR and using PRNT to reduce the proportion of false-positive results caused by cross-reactivity (Rabe et al., 2016), we were able to obtain more accurate diagnostic results.

The main limitation of this study was that in the MERG PWC cohort the number of samples submitted to all available tests at each blood sample collection point varied. Not all pregnant women participated in all sample collections. We do not believe that the adopted strategy—repeated cross-sectional assessments within the time strata of the cohort—distorted the results, since the opportunity for testing was not related to any specific criterion.

A limitation of the ZIP cohort study is the use of interval censoring, which is inherent to survival studies. In the present study, the median time between the last positive test and the documented seroreversion was 30 days (P25 = 28; P75 = 36), which may have overestimated the time to seroreversion by approximately 15 days.

## 6 Conclusion

This study has presented original findings previously unexplored in the literature regarding the time-dependent behavior of IgM, IgG3 antibodies, and neutralizing antibodies in response to ZIKV infection in pregnant women. Using two distinct analytical approaches - survival analysis and successive cross-sections, the results from both methods align consistently. In the survival analysis, the estimated median time to IgM seroreversion was 3.5 months. In successive cross-sectional assessments, the prevalence of IgM positivity began to decrease between the second and third months, with lower levels observed from the fourth month onward.

The data obtained concerning the probability of antibody detection over time are relevant given the need to understand this poorly documented dynamic. Therefore, it is necessary to conduct further research focused on this area to aggregate and solidify our knowledge. Consequently, further research in this area is essential to deepen and consolidate our knowledge. Our findings may also serve as a basis for developing strategies aimed at pregnant women, suggesting adjustments to testing protocols that identify the optimal testing windows and the most appropriate test type and interpretations, thereby strengthening maternal and child health surveillance.

## Ethical statement

This study was developed following approval by the Research Ethics Committee of the HUOC/PROCAPE Hospital Complex and has the Certificate of Presentation for Ethical Assessment (CAAE) No. 70198223.0.0000.5192 and a substantiated report No. 6.209.246 issued on July 31, 2023. The Research Ethics Committee of the IAM-Fiocruz-PE provided ethical approval for the MERG Pregnant Women’s Cohort (PWC) under CAAE: 5324.0816.4.0000.5190 and a substantiated report No 1.533.226 issued on May 6, 2016 and for the Zika in Infants and Pregnancy (ZIP) Cohort under CAAE: 5667.3616.3.2001.5190 and a substantiated report No 1.697.660 issued on 26 August 2016. In addition to having guaranteed the standard procedures recommended by Resolution MS/CNS 466/2012, informed consent was obtained from all participants prior to enrollment.

## Data statement

In accordance with the standards for good clinical research of the Research Ethics Committee of the Aggeu Magalhães / Fiocruz Research Center, data cannot be shared publicly because the dataset contains sensitive human subject data. Participants did not provide consent for public sharing of their data, and public availability would compromise patient privacy. De-identified data can be made available upon reasonable request from qualified investigators by contacting the Programa de Pós-Graduação em Ciências da Saúde (PPGCS) da Universidade de Pernambuco (UPE) at ppg.cienciasdasaude@upe.br

## CRediT authorship contribution statement

**Maria Valquíria de Medeiros Silva:** Writing – review & editing, Writing – original draft, Visualization, Supervision, Project administration, Methodology, Investigation, Formal analysis, Data curation, Conceptualization. **Ricardo Arraes de Alencar Ximenes:** Writing – review & editing, Writing – original draft, Visualization, Validation, Supervision, Resources, Project administration, Methodology, Investigation, Funding acquisition, Formal analysis, Data curation, Conceptualization. **Demócrito de Barros Miranda Filho:** Writing – review & editing, Writing – original draft, Visualization, Validation, Supervision, Resources, Project administration, Methodology, Investigation, Funding acquisition, Formal analysis, Data curation, Conceptualization. **Celina Maria Turchi Martelli:** Writing – review & editing, Writing – original draft, Visualization, Supervision, Resources, Project administration, Methodology, Investigation, Funding acquisition, Formal analysis, Data curation, Conceptualization. **Thalia Velho Barreto de Araújo:** Writing – review & editing, Writing – original draft, Visualization, Supervision, Resources, Project administration, Methodology, Investigation, Funding acquisition, Formal analysis, Data curation, Conceptualization. **Elizabeth Brickley:** Writing – review & editing, Writing – original draft, Visualization, Methodology, Formal analysis. **Juliana Menezes Soares de Souza Azevedo Fontes:** Writing – review & editing, Writing – original draft, Investigation, Conceptualization. **Gabriela Renata Neves Fulco:** Writing – review & editing, Writing – original draft, Investigation, Conceptualization. **Ana Carolyne de Carvalho Lucena Sá:** Writing – review & editing, Writing – original draft, Investigation, Conceptualization. **Marli Tenorio Cordeiro:** Writing – review & editing, Writing – original draft, Investigation, Conceptualization. **Priscila Mayrelle Da Silva Castanha:** Writing – review & editing, Writing – original draft, Investigation, Conceptualization. **Paula Alexandra dos Santos Oliveira:** Writing – review & editing, Writing – original draft, Supervision, Investigation, Conceptualization. **Ulisses Ramos Montarroyos:** Writing – review & editing, Writing – original draft, Visualization, Validation, Supervision, Resources, Project administration, Methodology, Investigation, Funding acquisition, Formal analysis, Data curation, Conceptualization.

## Declaration of competing interest

The authors declare that they have no known competing financial interests or personal relationships that could have appeared to influence the work reported in this paper.

## Data Availability

The authors do not have permission to share data.

## Appendix Supplementary materials

Image, application 1
