# Supplementary material for: Kinetics of neutralizing and binding antibody responses following Zika virus infection during pregnancy: A nested analysis of participants from the microcephaly epidemic research group (MERG) and Zika in infants and pregnancy (ZIP) cohorts
Source: Virus Res. 2026 Apr 5;368:199725. doi: 10.1016/j.virusres.2026.199725 (PMC13126471; doi:10.1016/j.virusres.2026.199725)
Supplement: Supplementary file 1 [file mmc1.docx]

**Glossary**

**Congenital Zika Syndrome (CZS):** It is characterized by a series of structural and functional abnormalities, which include, in addition to microcephaly, imaging, neurological, and ophthalmological alterations in newborns whose mothers were infected with ZIKV during pregnancy.

**Enzyme-linked immunosorbent assay (ELISA) test:** Laboratory assay that detects the presence of specific antibodies or antigens in a sample, using antigen-antibody binding and an enzymatic marker that produces a measurable signal.

**Immunoglobulin G 3** (**IgG3):** IgG subclass of antibodies that, due to its rapid production kinetics and short half-life, can be detected in serology as a marker of recent infection.

**Immunoglobulin M** (**IgM):** First class of antibodies produced by the immune system in response to an infection or antigen exposure. Due to its rapid production after initial exposure and transient presence in serum, IgM detection is widely used as a marker of recent or acute infection.

**PRNT (Plaque Reduction Neutralization Test):** Serological assay considered the gold standard for the detection and quantification of virus-neutralizing antibodies. It is based on the ability of these antibodies to inhibit infection of cells in culture, resulting in a reduction in the number of viral plaques formed.

**RT-PCR:** Molecular technique for the detection of viral nucleic acid by reverse transcription polymerase chain reaction (RT-PCR), allowing the detection and/or quantification of specific RNA sequences.

**Seroconversion:** Characterized by the laboratory detection of the appearance of specific antibodies against a given antigen in previously negative serum samples.

**Seropositive:** Refers to the detection of specific antibodies against a given infectious agent or antigen in serological tests.

**Seronegative:** Refers to the absence of detectable antibodies against a given infectious agent or antigen in serological tests.

**Seroreversion:** Characterized by the loss of detectable antibodies in blood samples that were previously positive against a specific antigen.

**Viremia:** period during which viral particles are present and circulating in the blood.
